# Potential Benefits of a Minimal Dose Eccentric Resistance Training Paradigm to Combat Sarcopenia and Age-Related Muscle and Physical Function Deficits in Older Adults

**DOI:** 10.3389/fphys.2021.790034

**Published:** 2021-11-30

**Authors:** Sara A. Harper, Brennan J. Thompson

**Affiliations:** ^1^Department of Kinesiology and Health Science, Utah State University, Logan, UT, United States; ^2^Sorenson Legacy Foundation Center for Clinical Excellence, Dennis Dolny Movement Research Clinic, Utah State University, Logan, UT, United States

**Keywords:** muscle function, resistance training, strength training, maximal strength, aging

## Abstract

The ability of older adults to perform activities of daily living is often limited by the ability to generate high mechanical outputs. Therefore, assessing and developing maximal neuromuscular capacity is essential for determining age-related risk for functional decline as well as the effectiveness of therapeutic interventions. Interventions designed to enhance neuromuscular capacities underpinning maximal mechanical outputs could positively impact functional performance in daily life. Unfortunately, < 10% of older adults meet the current resistance training guidelines. It has recently been proposed that a more “minimal dose” RT model may help engage a greater proportion of older adults, so that they may realize the benefits of RT. Eccentric exercise offers some promising qualities for such an approach due to its efficiency in overloading contractions that can induce substantial neuromuscular adaptations. When used in a minimal dose RT paradigm, eccentric-based RT may be a particularly promising approach for older adults that can efficiently improve muscle mass, strength, and functional performance. One approach that may lead to improved neuromuscular function capacities and overall health is through heightened exercise tolerance which would favor greater exercise participation in older adult populations. Therefore, our perspective article will discuss the implications of using a minimal dose, submaximal (i.e., low intensity) multi-joint eccentric resistance training paradigm as a potentially effective, and yet currently underutilized, means to efficiently improve neuromuscular capacities and function for older adults.

## Introduction

For older adults, interventions designed to enhance neuromuscular capacities underpinning maximal mechanical outputs could positively impact functional performance in daily life. Research has well established that relatively high levels of strength and power are important determinants for older adults’ functional capacities across a multitude of functional activities (Hanson et al., 2009; Reid and Fielding, 2012; Liu et al., 2014). Thus, resistance training (RT) is widely recommended as a key intervention for older adults to maintain activities of daily living (Martin Ginis et al., 2006), and to help combat age-related neuromuscular degenerations (Hurley et al., 2011; Mcleod et al., 2019). Not only is RT an important strategy to counteract age-related health and neuromuscular degenerations, it is the primary evidence-based intervention for addressing age-related losses of function and health given that muscle mass, strength, and/or power are associated with mobility (Reid and Fielding, 2012; McGregor et al., 2014; Ferrucci et al., 2016; Tieland et al., 2018), disease risk (Volpi et al., 2004; Wolfe, 2006; McLeod et al., 2016; Mcleod et al., 2019), and mortality (Metter et al., 2002; Newman et al., 2006). Unfortunately, older adults report barriers to RT compliance including time constraints and perceived difficulty (Fisher et al., 2017), with approximately 9.6% of older adults in the United States meeting the twice/week RT recommended guidelines (Kraschnewski et al., 2016). Therefore, alternative, perhaps even creative approaches, are necessary to improve widespread implementation and increase participation adherence for RT in older adults. Eccentric-based RT could be uniquely qualified as an effective and feasible “minimal” RT strategy that may result in greater exercise program adherence and widespread adoption by addressing some of the common barriers to training.

Eccentric RT exercise is characterized by high force, muscle overloading contractions that can induce substantial neuromuscular adaptations (Hoppeler, 2016; Molinari et al., 2019). It may be a particularly promising approach for older adults to efficiently improve muscle mass, strength, and functional performance. A recent investigation suggests that older adults retain remarkable capacity for adaptation to those of their younger counterparts (Quinlan et al., 2021). This capacity, along with the coupling of low energy cost to high force production (LaStayo et al., 2003), offer features to allow older adults to train to their relative physiological advantages. Due to its “metabolic efficiency” at a given workload, coupled with emerging evidence that suggests that lower intensity eccentric exercise can elicit improvements in physical function and muscle function (i.e., strength) (Johnson et al., 2018; Kay et al., 2020), this form of exercise could provide a much needed solution to help more older adults engage in, and benefit from, RT activities.

From a safety perspective, high force RT could have the potential to increase risk (e.g., soreness, risk of injury) without proper familiarization and training among older adults. Given that sedentary and/or mobility-limited older adults are less likely to meet the recommended RT guidelines (i.e., they are out of shape), the stimulus threshold necessary to induce adaptations in this population should be rather low (with a more limited, submaximal, stimulus requirement)—which aligns with the principal of diminishing returns for mobility improvements (Brahms et al., 2021). Given these rather novel RT concepts, our perspective article will discuss the potential implications of using a minimal dose, submaximal (i.e., low intensity) eccentric RT paradigm as a potentially effective, and yet currently underutilized means to efficiently improve maximal neuromuscular and functional capacities for older adults.

## Eccentric Resistance Training for Older Adults

### Benefits and Challenges of Resistance Training

In recent years, RT has become widely recognized as among the most effective means to combat the myriad of age-related ailments. Overwhelming evidence for the benefits of RT in counteracting numerous aging disorders has spawned recent review papers (Holm et al., 2015; Fisher et al., 2017; Mcleod et al., 2019; Tavoian et al., 2020) that have provided compelling arguments for RT to be used as a sort of “prophylactic” for aging (Fisher et al., 2017). These reviews present supporting evidence to substantiate their recommendation that RT should become a more central element in virtually all exercise position stands (e.g., professional and federal recommendations) (Mcleod et al., 2019; Tavoian et al., 2020).

Unfortunately, the low participation rate in older adults is, at least in part, influenced by specific barriers to participation that include burdensome time commitment and discomforts pertaining to rating of perceived exertion (RPE) (Schutzer and Graves, 2004; Lees et al., 2005; Justine et al., 2013). These barriers call into question current RT recommendations and their effective application for older adults. In fact, there are calls for a “minimal dose” RT approach for older adult programming to increase widespread participation for older adults who possibly stand to benefit the most from RT implementation (Byrne et al., 2016; Fisher et al., 2017). For instance, (Byrne et al., 2016) have suggested that the American College of Sports Medicine (ACSM) RT guidelines (e.g., 2–4 days/week at 1–3 sets of 8–12 repetitions across 6–12 major muscle groups) for older adults, “may not represent the most targeted and efficient means of improving the physiological impairments…associated with functional performance,” and that the logistical constraints, “limit the widespread adoption of the ACSM model as a pragmatic intervention.” Therefore, alternative approaches that are more conducive to real-world applications compared to ACSM RT guidelines may help improve widespread implementation and increase adherence for older adults (Byrne et al., 2016).

We discuss below a potential “alternative” approach that may resolve some of the barriers responsible for the poor RT participation of the older adult population. Namely, we provide our perspective on how multi-joint eccentric RT using submaximal training intensities in an overall minimal dose paradigm may be a promising, practical approach to yield beneficial effects for older adults.

### Promising Solution: An Eccentric-Based Resistance Training Model

Eccentric-based RT offers some unique physiological advantages compared to conventional RT. Specifically, eccentric exercise is highly metabolically efficient to the end that less perceived exertion (known as rating of perceived exertion; RPE) is experienced when performing the same amount of external workload as compared to concentric exercise (Reeves et al., 2009). Also, despite the relative lower RPE, eccentric exercise is ideally suited for eliciting an overloading of the muscle-tendon unit due to inherent muscle properties (including both active and passive components) that provide for high intrinsic muscle forces during eccentric contractions, which can result in substantial neuromuscular adaptations (e.g., hypertrophy, muscle strength) (Farthing and Chilibeck, 2003; Reeves et al., 2009; Roig et al., 2009). Applying these eccentric advantages toward a “minimal dose” RT approach could be particularly beneficial. Fortunately, the physiological advantages of eccentric exercise are especially well suited for implementing an effective minimal dose training model.

### Efficient Training Adaptations of Eccentric Resistance Training

Eccentric exercise can elicit substantial gains in muscle size, strength, and power, with a relatively low overall workload—and therefore, reduced training time—compared to concentric (Farthing and Chilibeck, 2003), or conventional RT (Reeves et al., 2009). Consequently, the efficiency of eccentric training in eliciting training adaptations yields an opportunity to achieve substantial gains, with less time and overall exertion (both perceived and real) invested in the exercise program.

Moreover, previous investigations have shown the presence of greater eccentric strength preservation associated with aging compared to concentric strength (Hortobágyi et al., 1995; LaStayo et al., 2003). This preservation of eccentric strength capacity (∼21% more versus concentric strength) (Roig et al., 2010), along with the coupling of low energy cost to relatively high force production (LaStayo et al., 2003) would allow older adults to train to their relative physiological advantages.

Combining the specific advantages of lowered RPE with the time/workload efficient adaptation response and relatively amplified eccentric strength in older adults, together, offers a promising paradigm for eccentric RT for overcoming major barriers for RT participation of older adults. Taken together, the physiological virtues of eccentric exercise provide a convincing argument for further investigating the use of a minimal dose eccentric RT approach in older adults.

### Eccentric-Based Resistance Training Equipment

As a result of the recent upsurge of positive research findings from eccentric training, eccentric-based RT equipment is quickly becoming more widely available. For example, more compact motorized units have exponentially increased in number due to cost efficient advances in computer-servomotor based technology, machines with variable cam designs that harness the eccentric phase, pneumatic-driven machines, and flywheels (which harness inertia to increase the eccentric overload phase). In fact, in addition to an increasing selection of standard flywheel training devices, a number of flywheel devices are now on the market that use smart electric motor technologies to provide more energy to the flywheel speed (i.e., increased load) specifically in the eccentric phase of the movement. One device that exhibits a number of favorable features for improved accessibility is a very small flywheel (2 lbs.), known as Handy Gym that can be purchased for under $1,000, is able to provide up to 220 lbs. of resistance, and can be used across a wide range of exercise activities.

**FIGURE 1:**
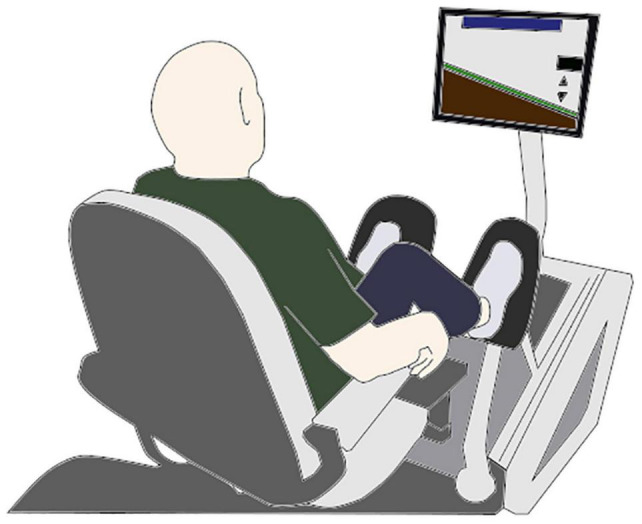
Eccentron machine illustration, a multi-joint eccentric RT approach. The machine uses a motor to drive the pedals toward the subject, unilaterally in an alternating manner. The force level produced by the subject is viewed in real time on a computer simulation, which presents a target for the subject to attempt to reach with each repetition. The movement primarily involves the muscles surrounding the knee and hip joints, and to a lesser degree the ankle.

Other equipment of interest includes a seated dynamometer known as an Eccentron (Figure 1), which is a negative resistance trainer that delivers a multi-joint isokinetic eccentric-based movement. This type of instrument has been used in research settings and offers the advantage of control over the velocity of the movement and, thus, is valuable from a research perspective because it can yield a standardized workload which can provide for high control and quantification of the variables comprising the RT routine (e.g., force, workload, volume, and velocity) (Tinwala et al., 2017).

In practice, the increased availability of a variety of eccentric-overload capable devices will help provide widespread opportunities for eccentric exercise implementation for the population at large. It is also noteworthy that specialized exercise instruments are not necessarily needed to generate an eccentric overload. Simple field techniques using basic equipment are also readily capable of eliciting an eccentric overload. For instance, one simple technique includes lifting (concentrically) a moderately heavy load with both limbs and lowering the load with only one limb.

An understanding of specific physiological adaptations and eccentric training programming nuances for older adults with the associated benefits would help empirically inform professional organizations when updating their guidelines as they continually adapt to the increasingly prevalent eccentric RT modality.

### Eccentric-Based Training: Using a Multi-Joint Training Model

#### Exercise Intensity

The vast majority of previous studies examining eccentric RT have implemented a moderate or high intensity training routine (using our definition of >50% of maximal eccentric strength and/or ≥12 on the Borg scale) (LaStayo et al., 2003, 2007, 2011, 2017; Dibble et al., 2006; Jacobs et al., 2014; Tanner et al., 2015; McKenzie et al., 2017; Suh et al., 2017; Briggs et al., 2018; Reidy et al., 2018; Gordon et al., 2019; Crane et al., 2020; Kumar, 2021). While the gains from these doses were indeed favorable, it is possible that such loads are more excessive than what is needed to elicit adaptations from eccentric RT since eccentric contractions inherently create high internal muscle-tendon forces, even without performing near maximal contractions (Hody et al., 2019).

To our knowledge, only two investigations have used a relatively low percentage of maximal eccentric strength for their multi-joint eccentric RT routine in older adults on an Eccentron machine (Johnson et al., 2018; Kay et al., 2020). Johnson et al. (2018) eccentric training intensity progressed from 20 to 50% of maximal eccentric strength (after familiarization) and elicited significant improvements in physical function measures including the 30-s repeated chair stand (partial η^2^ = 0.51; large effect size) and the Timed Up and Go (TUG) test (partial η^2^ = 0.69; large effect size) in older adults (63.5 years). In addition, (Johnson et al., 2018) trained older adults (67.1 years) on an Eccentron at 50% of maximal eccentric strength and reported substantial improvements in mobility (improved TUG time, −12 ± 8.9%, Cohen’s *d* = 1.34; large effect size), eccentric strength (58.8 ± 39.9%, *d* = 1.80; large effect size), and vastus lateralis thickness (9.8 ± 1.3%, *d* = 2.54; large effect size). Collectively, these findings provide evidence that lower intensity eccentric RT interventions (modeling a minimal dose routine) can elicit significant adaptations and positive functional outcomes for older adults. However, more work needs to be done elucidating the effects of such routines on soreness and inflammatory markers in older adults.

#### Exercise Session Duration

Exercise session durations for multi-joint eccentric training have varied considerably within the broad (all adult ages) literature from a more minimal dose (e.g., three/min sessions) (Gordon et al., 2019; Crane et al., 2020) up to 30 min/sessions (Dibble et al., 2006; Suh et al., 2017; Lim and Lee, 2018; Kim et al., 2019). While a three/min exercise session may represent a minimal training approach, this previous work was conducted in young adults (Gordon et al., 2019; Crane et al., 2020). Thus, it is unknown how older adults will respond to a shorter duration exercise session. Only two investigations in older adults have been conducted with relatively short exercise sessions with (Johnson et al., 2018) starting at three/min and progressing up to 10/min exercise sessions, and (Kay et al., 2020) starting at five/min and progressing up to 10/min exercise sessions. In both studies, there were substantial improvements from these low exercise session duration training routines (Johnson et al., 2018; Kay et al., 2020). Thus, the minimal duration eccentric exercise approach shows some promising potential; however, additional work is needed to assess the minimal dose exercise session framework in older adults.

#### Exercise Training Frequency

Commonly investigated training frequencies in eccentric training studies vary from two (Johnson et al., 2018, 2019; Kim et al., 2019; Kay et al., 2020; Kumar, 2021) and three exercise sessions per week (Dibble et al., 2006; Jacobs et al., 2014; Tanner et al., 2015; Lim and Lee, 2018; Reidy et al., 2018), with (Byrne et al., 2016) reporting that, “a frequency of twice weekly can produce significant improvements in power and physical function. A compelling case for lower training session frequency is that a *“less is more”* exercise session frequency was found to be much more preferred among older adults (Foley et al., 2011). For instance, although training 3 days/week has been a commonly used frequency in a multitude of training studies (LaStayo et al., 2003, 2011, 2017; Dibble et al., 2006; Jacobs et al., 2014; Tanner et al., 2015; Lim and Lee, 2018; Reidy et al., 2018), only 1% of older adults prefer exercise training 3 days/week (Foley et al., 2011). In young adults, (Crane et al., 2020) compared 1 day/week versus 3 days/week with an equalized workload on a multi-joint eccentric training routine and found that there was no statistical advantage on the improvements for the three versus 1 day/week training condition. Future research should be conducted on varying exercise training frequency conditions among older populations to determine whether similar results are obtained for this population.

#### Exercise Progression

For sedentary and/or mobility-limited older adults, exercise progression is an important component in any RT program. For this population, (LaStayo et al., 2003) conducted eccentric RT research using the RPE scale, in which the training started at “very, very light” (7 out of 20 on the Borg scale) and progressed to “very light” (9 out of 20) and “fairly light” (11 out of 20) after 3 weeks of training (LaStayo et al., 2003, 2007, 2011, 2017). Their RPE-based approach could be a practical model to use for eccentric RT progression in older adults. Eccentric RT progression approaches could also be implemented using the relative maximal eccentric strength method as a measure of gauging intensity and its progression. As eluded to previously (Johnson et al., 2018) eccentric training intensity progressed from 20 to 50% of maximal eccentric strength (after familiarization) in a study that used the relative intensity method. Duration of exercise training can also progress throughout the exercise training program with familiarization training starting at three min/session (Dibble et al., 2006; LaStayo et al., 2007, 2011, 2017; Jacobs et al., 2014). In comparison, (Johnson et al., 2018) began at 5 min session and progressed up to 10 min sessions (Johnson, 2018), demonstrating how programs could increase from shorter to longer durations over the course of several weeks. However, overall the literature is limited regarding these training variables in an eccentric RT context. Collectively, intensity, duration, frequency, and progression of training research is needed to optimize the training stimulus for older adults in a manner that meets the training stimulus threshold for adaptations, but does not induce excessive drawbacks from a soreness, injury risk, or overtraining aspect.

#### Addressing Barriers for Adherence

A minimal eccentric RT dose approach may address commonly reported barriers for adherence among older adults who are not meeting the current recommended RT guidelines. Specifically, the minimal RT dose approach could address challenges associated with frequency of RT sessions (e.g., reduce the frequency of the need to travel to the gym, where transportation is difficult), overall time constraints, and discomfort (e.g., exertion). Also multi-joint, lower body eccentric RT has the potential to improve intervention adherence, as for example, (LaStayo et al., 2007) showed that ≥90% of their older adult subjects completed 80.5% of exercise sessions throughout a 6 month eccentric RT program, and a 99% completion rate has been reported for both 6 week (Kay et al., 2020) and 8 week (Johnson et al., 2019) multi-joint eccentric RT interventions. While there appears to be a high potential for muscle-related gains from minimal dose eccentric RT paradigms in older adults, additional work is needed to assess adherence for longer intervention lengths (e.g., >12 weeks) and to more directly compare primary training factors including various levels of intensity, duration, and frequency in the context of minimal dose eccentric RT.

While a considerable amount of the research literature has focused on investigating the effects of eccentric RT using a single joint, isolated muscle group training model, such as with knee extension or elbow flexion exercise movements (Roig et al., 2009), multi-joint exercises offer some important advantages for training in a minimal dose model. A multi-joint training approach minimizes the peak forces on a single-joint while maximizing the benefits of the negative work approach (Mueller et al., 2009). Moreover, training more than one joint in a single exercise increases the training effect by involving more muscles, which is a more efficient way to train as it alleviates the need to individually target multiple smaller muscles. This can lead to reduced training time due to the overall reduced number of sets required to work the major muscle groups. For these reasons, using multi-joint training is a desired feature of a “minimal” RT routine.

Previous research has investigated the use of multi-joint eccentric-based RT in older adults (LaStayo et al., 2003, 2007, 2011, 2017; Mueller et al., 2009, 2011; Jacobs et al., 2014; Tanner et al., 2015; Johnson et al., 2018, 2019; Reidy et al., 2018; Kay et al., 2020; Kumar, 2021). Two eccentric RT routines that may generally be considered “minimal dose” in terms of the training features such as with intensities of 30–50% of maximal eccentric strength (Johnson et al., 2018; Kay et al., 2020). Notably, as eluded to previously, (Kay et al., 2020) conducted a multi-joint lower body RT intervention for older adults, trained up to 10 min/session (20 min/week) and found substantial improvements after training and (Johnson et al., 2018) also had relatively short duration exercise sessions (up to 10 min/session, 20 min/week) and revealed significant improvements after training. These are promising outcomes since the magnitude of these reported changes tends to be associated with training interventions exhibiting a more substantial weekly training dose. Thus, these studies provide supporting evidence that a minimal, multi-joint eccentric RT may lead to mobility and functional improvements.

However, limitations exist in the literature specifically for well controlled studies that examine a comprehensive set of key functional, physiological, and qualitative outcomes and dosing-related (e.g., exercise session duration, intensity, and frequency) comparisons in the context of a minimal dose, multi-joint training paradigm in older adults. Future research should consider making direct varied dose comparisons among these numerous training factors to help clarify more precisely a minimal dose threshold at which meaningful gains may still be produced. Such research would be instrumental in helping to guide and provide minimal dose guidelines regarding the amount of training dose necessary to elicit neuromuscular and functional gains.

#### Potential Benefits of Minimal Dose Eccentric Resistance Training

There is a high potential for muscle-related gains from minimal dose eccentric RT, especially for sedentary, older adults who are commonly far below their maximal neuromuscular function potential. For instance, (LaStayo et al., 2003) have suggested that eccentric RT, “*via* negative work, cannot only be performed with tolerable low-to-moderate effort in an at-risk elderly population, but can also increase muscular size and strength dramatically.” Minimal dose eccentric RT may also be effective at increasing muscular strength, power, mass, and physical function (Johnson et al., 2018; Kay et al., 2020), but with the advantage of having more favorable soreness and inflammation responses compared to higher intensity protocols, which would increase the tolerability of the training routine (Tse et al., 2015). These factors offer support for investigating the use of minimal dose eccentric RT paradigms in older adults.

#### Issues and Limitations With the Proposed Approach

While there are a number of potential benefits to the proposed RT model, we acknowledge there are some potential drawbacks that must be considered in order for such an approach to be effectively managed. First, although eccentric RT indeed appears to be an efficient training approach, this form of exercise increases the likelihood of delayed onset muscle soreness (DOMS) through contraction-induced muscle damage, which also leads to a short-term decline in muscle function (i.e., depressed maximal muscle strength and power capacity in the 24–72 h post exercise). Concerns for exercise-induced muscle damage (and its consequences) that may be associated with higher intensity eccentric RT further supports the need for investigating low intensity eccentric RT paradigms in older adults (Tse et al., 2015). In support of lower intensity eccentric RT, induced changes over time may elicit a muscle damage protective effect (Chen et al., 2021). The minimized muscle damage feature of lower intensity eccentric RT is a very important part of improving the tolerance of the RT exercise routine in older adults due to soreness-related discomforts. Therefore, markers of muscle damage (e.g., soreness) should be closely monitored during the RT routine, and principles of gradual progression should be employed when designing and implementing the program.

While work from Johnson et al. (2018) and Kay et al. (2020) provides encouraging data regarding a minimal dose eccentric RT approach, there are aspects that have yet to be explored. For example, rigorous dose comparison work is necessary to determine the relative performance of the proposed minimal dose. One limitation the proposed eccentric model is the inclination for a rather specialized piece of equipment in order to do a full range of eccentric-accentuated RT exercises. These devices can be rather costly and may require specialized knowledge to use. However, as mentioned in section “Eccentric-based RT equipment” above, the accessibility and practicality of these devices is rapidly evolving to the point that there are now devices on the market that are relatively inexpensive, lightweight, portable, and offer a wide range of resistance exercises. Given the recent interest in eccentric exercise, and the technological advances, such devices are likely to increase in number in the near future, increasing the overall accessibility and utility of this form of exercise. To this point, it would be highly useful for future research to examine the effectiveness of such devices on measures of physical and muscle function as well as the adherence and preferability factors in older adult populations.

Finally, it should be noted that this proposed RT model is not intended to be a replacement for the currently established RT guidelines. It is merely an alternative approach that may be suitable for a certain proportion of the population, that currently does not, or perhaps never will, adhere to the current guidelines due to barriers associated with them. It is of course recommended, for those who will, to adhere to the current RT recommendations. However, only 10% meet the guidelines, and so the minimal dose eccentric RT model is presented here as a potentially effective means that may help to get more people to gain at least some of the benefits of RT, even if it does not provide the maximally available adaptation response or the maximal benefits, the argument here is that some benefit is better than none.

## Conclusion

The ability of older adults to optimally perform activities of daily living is often limited by the ability to generate high mechanical outputs. Therefore, assessing and developing maximal neuromuscular capacity is essential for determining age-related risk for functional decline, elucidating the effectiveness of therapeutic interventions, and improving health and functional capacities. Unfortunately, the vast majority (∼90%) of older adults do not meet RT guidelines (Kraschnewski et al., 2016) despite the many demonstrable benefits of RT for older adults.

Our perspective here shows how the implementation of a minimal dose eccentric-based RT program offers several advantages for overcoming common barriers to RT participation in older adults while still potentially achieving significant gains in neuromuscular function. The outcomes of such a paradigm could lead to overall health and functional living improvements through heightened exercise tolerance and participation in a feasible RT program. An important, yet perhaps underappreciated, goal is to get a higher proportion of the older adult population to participate in RT so that they may gain some of the benefits from this important counter-aging activity. A minimal RT dose approach seems to be a promising solution toward getting people to participate in RT, who otherwise would not do so. We have proposed a potential RT model that includes a minimal dose, submaximal (i.e., lower intensity) multi-joint eccentric paradigm, which could be a potentially effective means to efficiently improve maximal neuromuscular capacities for older adults including the potential benefits for improving daily function.

## Data Availability Statement

The original contributions presented in the study are included in the article/supplementary material, further inquiries can be directed to the corresponding author/s.

## Author Contributions

SH and BT conceived the research perspective and wrote the initial draft, critically reviewed the manuscript. Both authors contributed to the refinement of the final manuscript.

## Conflict of Interest

The authors declare that the research was conducted in the absence of any commercial or financial relationships that could be construed as a potential conflict of interest.

## Publisher’s Note

All claims expressed in this article are solely those of the authors and do not necessarily represent those of their affiliated organizations, or those of the publisher, the editors and the reviewers. Any product that may be evaluated in this article, or claim that may be made by its manufacturer, is not guaranteed or endorsed by the publisher.

## Abbreviations

RT: resistance training
RPE: rating of perceived exertion
ACSM: American College of Sports Medicine
TUG: Timed Up and Go
DOMS: Delayed-Onset Muscle Soreness.
